# Identifying established human placental markers of schizophrenia in rodents after gestational ∆9-tetrahydrocannabinol exposure^†^

**DOI:** 10.1093/biolre/ioaf191

**Published:** 2025-08-19

**Authors:** Andrea M Kocsis, Enzo Perez-Valenzuela, Mar Rodríguez-Ruiz, Mohammed H Sarikahya, Anubha Dembla, David R C Natale, Steven R Laviolette, Daniel B Hardy

**Affiliations:** Department of Physiology and Pharmacology, London, Ontario, Canada; Department of Obstetrics and Gynaecology, London, Ontario, Canada; Schulich School of Medicine and Dentistry, London, Ontario, Canada; Children’s Health Research Institute, London, Ontario, Canada; Western University, London, Ontario, Canada; Department of Anatomy and Cell Biology, London, Ontario, Canada; Schulich School of Medicine and Dentistry, London, Ontario, Canada; Western University, London, Ontario, Canada; Department of Anatomy and Cell Biology, London, Ontario, Canada; Schulich School of Medicine and Dentistry, London, Ontario, Canada; Western University, London, Ontario, Canada; Department of Anatomy and Cell Biology, London, Ontario, Canada; Schulich School of Medicine and Dentistry, London, Ontario, Canada; Western University, London, Ontario, Canada; Department of Physiology and Pharmacology, London, Ontario, Canada; Schulich School of Medicine and Dentistry, London, Ontario, Canada; Western University, London, Ontario, Canada; Department of Obstetrics and Gynaecology, Queen’s University, Kingston, Ontario, Canada; Department of Anatomy and Cell Biology, London, Ontario, Canada; Schulich School of Medicine and Dentistry, London, Ontario, Canada; Western University, London, Ontario, Canada; Department of Physiology and Pharmacology, London, Ontario, Canada; Department of Obstetrics and Gynaecology, London, Ontario, Canada; Schulich School of Medicine and Dentistry, London, Ontario, Canada; Children’s Health Research Institute, London, Ontario, Canada; Western University, London, Ontario, Canada

**Keywords:** cannabis, placenta, schizophrenia (SCZ), Δ9-tetrahydrocannabinol (THC), fetal growth restriction, DOHaD

## Abstract

Placental complications resulting in fetal growth restriction have been associated with dysregulated placental gene expression tied to an increased risk of schizophrenia. In rat offspring, it has been demonstrated that ∆9-tetrahydrocannabinol exposure in pregnancy results in fetal growth restriction and schizophrenia-like phenotypes (*e.g*., decreased pre-pulse inhibition of the acoustic startle response). However, it remains elusive if prenatal ∆9-tetrahydrocannabinol exposure induces this schizophrenia signature of placental gene expression. Therefore, our objective was to determine if these established predictive markers of schizophrenia are altered in a preclinical model of gestational oral ∆9-tetrahydrocannabinol exposure in rodents. We observed significantly reduced fetal weights in male and female prenatal ∆9-tetrahydrocannabinol-exposed offspring in the absence of maternal pregnancy outcomes. Placentae from ∆9-tetrahydrocannabinol-exposed males and females revealed altered expression of genes previously identified in human transcriptomic datasets of schizophrenia (i.e., *Furin, Rccd1,* and *Atp5mk*), with some expression changes being sex-specific (i.e., *Eif5, Rps10, Vps33b,* and *Iqgap1*). A subset of these genes were found differentially expressed in human BeWo cells exposed to ∆9-tetrahydrocannabinol. Targets were next examined in the adult rodent (postnatal day70) brain, and a subgroup of these genes (i.e., *Furin*, *Rps10*, and *Rccd1*) were increased concomitant with schizophrenia-like behavior (*e.g.*, decreased pre-pulse inhibition). We further detected ∆9-tetrahydrocannabinol-induced upregulation of FURIN in patient-derived cerebral organoids, an effect observed in both control and schizophrenia cell lines. Collectively, these findings demonstrate prenatal ∆9-tetrahydrocannabinol exposure can lead to altered gene expression in established prioritized markers of schizophrenia in the placenta in both animal and human models.

## Teaser

Prenatal THC exposure led to alterations in the expression of established prioritized markers of schizophrenia in placenta and brain models.

## Graphical Abstract

**Graphical Abstract:**
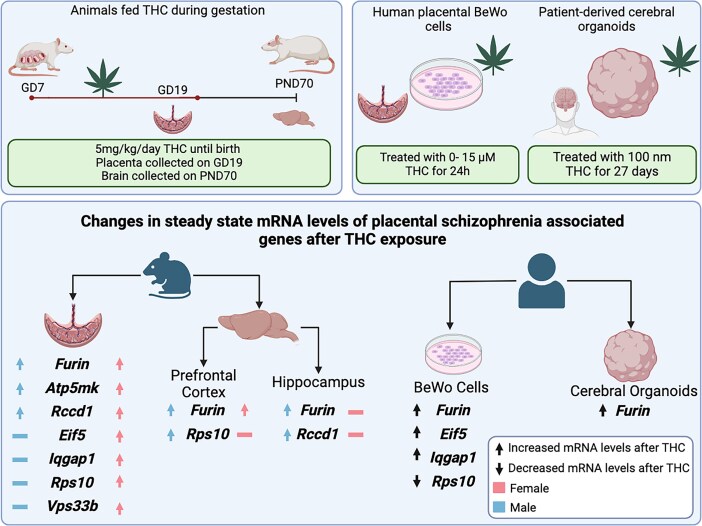

## Introduction

Cannabis use in the pregnant population has been increasing [1–3], with an estimated 5% of pregnant women reporting using cannabis daily in Canada [4]. However, the prevalence varies greatly depending on the method of detection and population being studied, with one study reporting 36% of pregnant women in Inuit populations using cannabis based on self-reports [5]. The most commonly reported purposes of cannabis use in pregnancy are to aid with anxiety, nausea, and insomnia [4]. However, despite this increase in consumption, the negative effects of prenatal cannabis are becoming more apparent [6]. Evidence suggests that exposure to cannabis in pregnancy increases the risk of poor fetal outcomes, such as fetal growth restriction (FGR) [7], which can lead to negative neurocognitive outcomes later in life (i.e., schizophrenia [SCZ]) [8, 9]. Moreover, it is postulated that ∆9-tetrahydrocannabinol (THC), the major psychoactive component of cannabis, could be a contributor to this increased risk of FGR [10, 11].

THC is a phytocannabinoid that can bind to the receptors of the endocannabinoid system (ECS): cannabinoid receptor 1 (CB_1_R) and cannabinoid receptor 2 (CB_2_R) [12]. As these receptors are present in central and peripheral tissues, the ECS exerts an influence on physiological processes ranging from placental development to cognition [13, 14]. Given its role in neurodevelopment, multiple studies indicate that early THC exposure correlates with an elevated risk of SCZ [15, 16], a disorder characterized by difficulties distinguishing reality and impaired cognitive function.

It is postulated that SCZ has neurodevelopmental origins, where a misconstruction of neural circuitry in utero reveals its consequent deficits later in life as these circuits become fully functional and utilized [17]. Specifically, mounting clinical evidence indicates that in utero cannabis exposure also confers adverse neuropsychiatric outcomes [18, 19], including psychotic-like behavior in children and young adults [19–21]. As clinical studies can be confounded by socioeconomic status and the use of other drugs, preclinical studies have been employed to help elucidate the specific effects of THC alone on SCZ risk. Gestational exposure to THC in pregnant rats has been demonstrated to lead to FGR and disrupt neuronal activity in the prefrontal cortex (PFC) and hippocampus (Hipp) [22, 23]. These disruptions were coupled with anxiety [24], cognitive defects, and decreased pre-pulse inhibition (PPI) [22, 23] in rats, which are similar to traits confirmed in humans with SCZ [25, 26].

While the consequences of THC exposure on the nervous system are well characterized, its effect on placentation remains understudied. Gestational THC exposure in rodents results in altered fetal–placental weight ratio, vascular defects in the placenta, and a lower expression of the placental glucose transporter, Glut1 [10]. Collectively, these findings, combined with clinical data, indicate that THC leads to placental insufficiency, culminating in FGR [27, 28]. These FGR outcomes are associated with impairment in neurodevelopmental health, leading to deficits in cognitive function [29], and an increased risk of neurodevelopmental diseases, including SCZ [8, 9, 30, 31].

A mechanistic framework linking placental development and neuropsychiatric disorders centers around the placenta–brain axis [32]. The placenta is responsible for the supply of nutrients to the fetal brain, and production of neurotransmitters, which is required for normal brain development [32]. Therefore, alterations in the placental gene expression affecting these processes have been linked to poor neurodevelopmental outcomes (*i.e*., SCZ) [33, 34]. Recent genome-wide and transcriptome-wide association studies have investigated genome or transcriptome variants and single-nucleotide polymorphism associated with SCZ in pregnancies with early life complications [35, 36]. From those studies, it was found that placentae from complicated pregnancies (i.e., those exhibiting FGR) led to a signature of SCZ-associated genes that were absent in normal pregnancies. Approximately 500 genes were expressed in the placenta correlated with SCZ outcomes, with ~20 prioritized genes being linked to both FGR and SCZ [36]. While THC can result in placental-insufficiency-induced FGR [10], it is unknown if THC can induce the differential gene expression associated with FGR and SCZ [35, 36]. Based on previous studies indicating links between prenatal THC exposure and SCZ [22–24], we hypothesize that these steady-state mRNA human placental markers of SCZ associated with FGR are also altered in our preclinical model of gestational THC exposure in rat placentae and in human models of the placenta and developing brain (*e.g*., cerebral organoids).

## Methods

### Edible THC rat model

All animal experiments were performed in accordance with the Canadian Council of Animal Care, following the ARRIVE guidelines. Experiments were performed as stated in the Animal Use Protocol (AUP no. 2023-129) approved by the Western University Animal Care Committee. Rats were obtained from Charles River on gestational day 5 (GD5) and given 2 days of acclimatization to the new environment and Nutella. THC was administered after day 6 given THC exposure prior to this time point can impair implantation in the rat [37].

Animals were kept at 22°C with a 12 h–12 h light–dark cycle and food and water ad libitum. Starting on GD7, pregnant rat dams ($n=7$ dams/group) were exposed to either THC in Nutella at 5 mg/kg or plain Nutella (control) from GD7-22. This route and concentration of THC have been demonstrated to lead to ~15–20 ng/mL THC in maternal circulation at gestational days 14 and 16, and ~30–50 pg/mL THC in placental and fetal tissues [38]. In human studies, it has been demonstrated that the physiological range of measured plasma concentrations can be from 60 to 200 ng/mL [39–41], indicating this dose of edible THC is similar to what is observed in humans. In pregnant rat dams, oral administration of this dose of THC did not impact litter size [42]. Rats were fed Nutella (treatment or vehicle) once daily until GD19.5, just before birth. Maternal measures (i.e., weight gain and food intake) were taken daily as previously published [10, 43]. On GD19.5, the first cohort of animals were sacrificed, and placentae were collected from each dam and placed in RNA later™. Fetal tail tissue was also collected from each corresponding pup and flash-frozen for SRY genotyping to confirm the sex of the offspring.

The second cohort of animals followed the same protocol but continued to be treated with THC until GD22 (birth). After weaning (post-natal day [PND]21), offspring from this cohort were housed at 22°C with a 12 h–12 h light–dark cycle and food and water ad libitum. Same-sex offspring were housed together in groups of three or four. The offspring from this cohort matured until PND70 (adulthood) when PPI of the acoustic startle response was assessed in animals. At this time point, adult animals were sacrificed, the Hipp and PFC were collected, and tissues were flash-frozen.

### Pre-pulse inhibition testing

PPI testing at PND70 ($n=3\hbox{--} 4$/treatment/sex, each offspring derived from distinct dams) was performed as previously described [44]. This point was selected since rodent studies have found SCZ-like behavior at a similar time point when exposed to THC in adolescence [44]. Furthermore, PND70 would be considered early adulthood, which is similar to when SCZ symptoms manifest in humans [45, 46]. Two days prior to PPI testing (day 1), animals were placed and allowed to acclimatize to the startle box for 5 min with 68 dB of background noise. The next day (day 2), animals were tested with an input/output function with 11 startle pulses ranging from 65 to 115 dB, increasing by 5 dB each time (20 ms duration, 1 min inter-trial interval). On day 3, PPI testing was performed. Testing was separated into two blocks. Block one consisted of 10 startle pulses at 110 dB lasting for 20 ms at 15–20 s intervals with 15–20 s inter-trail intervals. Block two consisted of pulses of 74, 77, 80, 83, or 86 dB (played at 20 ms intervals) before a loud pulse (110 dB) with interstimulus intervals of 100 ms. Ten trials at each pre-pulse volume were performed with pseudorandomized trials. To calculate %PPI the following formula was used: %PPI = 100 − [100 × (startle amplitude of pre-pulse + pulse trials/startle amplitude of pulse alone trials)].

### Rat Sex-determining Region Y (Sry) genotyping (PCR)

Frozen fetal rat tails were collected and underwent DNA extraction. PCR was then used to detect the male-specific *Sry* gene to determine the sex of the fetal pups since visual sexing was difficult at GD19.5. DNA was isolated and amplified using the REDExtract-N-AMP Tissue PCR Kit (Sigma-Aldrich), using the manufacturer’s instructions for DNA extraction. Amplification of the *Sry* gene used the forward sequence 5′- GGC TCA ACA GAA TCC CAG CA-3′ and reverse sequence 5′-TAG CCC AGT CCT GTC CGT AT-3′ as previously published [47]. PCR fetal tail products were run alongside DNA collected from adult male and female tissues collected from previous cohorts using the same protocol to act as controls for comparison.

### BeWo cell culture

To model human placental exposure to THC, unsyncytialized human trophoblastic placenta cells (BeWo cells) were used and purchased and utilized (ATCC no.: CCL-98). BeWo cells have been widely used as an in vitro model for cannabinoid effects on the placenta [48–50]. Cells were grown and treated as previously published [50]. Cells were cultured at 37°C and 5% CO_2_/95% atmospheric air in a cell culture incubator. Cells were grown in 75 cm^2^ flasks in F-12 K nutrient medium (Gibco) with 10% fetal bovine serum (Gibco) and 1% P/S (Penicillin/Streptomycin). Cells were plated on a 12-well plate with $2\times{10}^5$ cells per well. Prior to treatment, cells were allowed to seed for 24 h with 12 K nutrient medium. Cells were then exposed to media with 0 μM, 3 μM (940 ng/mL), or 15 μM (4700 ng/mL) of THC (dissolved in a final concentration of 0.1% ethanol, Cayman Chemicals, Ann Arbor, MI). These concentrations were selected based on previous research that demonstrated that exposure to 3–15 μM of THC did not cause a significant effect on the proliferation of these cells [48, 50, 51]. This study was designed to test the upper limit of exposure based on previous studies, as well as a 5× reduced concentration. Vehicle cells were treated with media with 0.1% ethanol. Cells were exposed to treatment for 24 h, then were collected and prepared for qPCR analysis.

### Induced pluripotent stem cell collection and maintenance

Twelve human-induced pluripotent stem cells (iPSCs), six from healthy donors and six from individuals diagnosed with SCZ were obtained from NIMH Repository & Genomics Resource, through Rutgers Infinity Biologix. To obtain the iPSC lines, study participants were consented and enrolled, dermal fibroblasts were collected, and iPSC cell lines were generated at Massachusetts General Hospital as part of an NIMH/NHGRI Center of Excellence in Genomic Science grant (P50MH106933). The Neurobank PI is Roy Perlis, M.D., MSc; key MGH co-investigators included Hannah Brown, M.D., J. Niels Rosenquist, M.D., Ph.D., Steven Sheridan, Ph.D., and Jennifer Wang, Ph.D. The CEGS co-PIs are Isaac Kohane, M.D., Ph.D. and Roy H. Perlis, M.D., MSc. Donors’ demographic information is provided in Table 1.

**Table 1:** Induced pluripotent stem cell subject demographic information

NIMH sample ID	Diagnosis	Sex	Race	Age (years)
MH0185913	CONTROL	M	White	26
MH0185983	CONTROL	M	White	29
MH0185984	CONTROL	M	White	29
MH0185865	CONTROL	F	White	24
MH0185916	CONTROL	F	White	25
MH0185905	CONTROL	F	White	33
MH0185897	SCZ	M	White	27
MH0185900	SCZ	M	White	31
MH0185975	SCZ	M	White	28
MH0185954	SCZ	F	More than one	34
MH0185964	SCZ	F	Asian	43
MH0185958	SCZ	F	White	56

Feeder-free iPSC cell lines were maintained in 4% oxygen in mTeSR1 medium (Stem Cell Technologies, CAT no. 85850) on Matrigel (Corning, CAT no. 354277) coated 6-well plates according to Stem Cell Technologies Maintenance of Human Pluripotent Stem Cell Technical Manual (Stem Cell hPSC Manual) (STEMCELL Technologies Inc. 2019). Cells were checked for potential differentiation events and were passaged using Gentle Cell Dissociation Reagent (StemCell, CAT no. 07174) when colonies were ~70% confluent.

### Organoid generation and treatment

Generation of human cerebral organoids was performed, as previously published [52], by keeping iPSC cells in culture for ~2 weeks after thawing and received a pre-treatment 2 days prior to differentiation. Pre-treatment consisted of growth factors that have been previously shown to promote broad telencephalic organoid competency and to enhance the formation of well-structured cortical organoids [53] at the following concentrations: Bone Morphogenetic Protein 4 (BMP4) = 0.1 ng/mL, Transforming Growth Factor β-1 (TGFβ-1) = 0.1 ng/mL, TGFβ-3 = 1.0 ng/mL, and ACTIVIN A = 15 ng/mL (all purchased from Stem Cell Technologies). Cerebral organoids were generated using the STEMdiff Cerebral Organoid Kit (Stem Cell Technologies, CAT nos. 08570 and 08571). To minimize batch differences, a protocol based on Lancaster and Knoblich was utilized [54]. Briefly, on day 0 of organoid generation (embryoid body [EB] formation), iPSCs were lifted using Gentle Cell Dissociation Reagent and a single-cell suspension was created by gently pipetting. Following centrifugation and resuspension, 100 μL of cell suspension (9000 cells/well) were plated in each well of a 96-well round-bottom ultra-low attachment plate (Corning, CAT no. 7007) with EB formation medium containing 10 μM rho-kinase inhibitor (StemCell, CAT no. 72302), and subsequently cultured in 5% CO_2_ in air. On days 2 and 4, 100 μL of EB formation medium was added to each well and on day 5 EBs were transferred to a 24-well ultra-low attachment plate (Corning, CAT no. 3473) containing Induction Medium. On day 7, organoids were transferred to an Organoid Embedding Sheet (StemCell, 08579), and cold Matrigel was added dropwise onto each EB. To polymerize the Matrigel, the plate was placed in the incubator at 37°C for 30 min. Upon removing the plate from the incubator, EBs were washed from the embedding surface into 6-well plates containing Expansion Medium. On day 10, all expansion medium was removed and replaced with 3 mL/well of maturation medium. The plates containing organoids were placed on an orbital shaker and incubated in 5% CO_2_ in air at 37°C. Following day 10, maturation medium was changed every 3 days, and, starting at day 40, Cultrex Reduced Growth Factor Basement Membrane Extract, Type 2 (R&D Systems, CAT no. 3536-005-02) was added to the maturation media at a 1:50 dilution to promote the formation and maintenance of a thick laminin-rich basement membrane, necessary for cortical plate formation [55]. Organoids were treated with THC 100 nM or vehicle (DMSO 0.1%) for 27 days, starting at day 3 up to day 30 of organoid generation, with medium changes performed every other day. Treatment doses and timings were based on Ao et al. [56], as it was determined that this dose did not cause premature organoid death over chronic THC exposure. After day 30, organoids were maintained without treatment and collected for qPCR on day 180. This resulted in four groups: vehicle control (VEH CTRL), THC control (THC CTRL), vehicle SCZ (VEH SCZ), and THC SCZ.

### RNA extraction and qPCR

Rodent tissue, human BeWo cells, and human cerebral organoids underwent total RNA extraction. For rodent placentae, the whole tissue underwent RNA extraction. In rodent brain, following PPI, all animals received an overdose of sodium pentobarbital (240 mg/kg intraperitoneal [*i.p*.] Euthanyl), and while under deep anesthesia, were sacrificed, brains rapidly removed, flash frozen on dry ice, and stored at −80°C. Coronal sections (100 μM) containing PFC and Hipp were cut with a cryostat tissue slicer (CM 1850) at −25°C and slide mounted for punchouts. These brain areas were collected for analysis as they are areas that are heavily implicated in SCZ [57, 58]. PFC brain sections were obtained from AP: +2.30 mm to +2.95 mm from Bregma, and Hipp brain sections were obtained from AP: −5.6 mm to −6.0 mm from bregma (The Rat Brain in Stereotaxic Coordinates, 6th Edition). The extraction of RNA was performed using Trizol and chloroform (Sigma Aldrich). RNA at a concentration of 2 μg was converted to cDNA using a high-capacity cDNA reverse Transcription Kit (Thermo Fisher Scientific; Applied Biosystems) following the manufacturer’s instructions. Of the 20 placental genes prioritized in the previous literature, 20 of the most differentially expressed genes, associated with FGR [35, 36], were selected to be tested in the prenatal THC-exposure rodent placenta. Genes associated with FGR were chosen as given THC-exposed offspring exhibit both FGR and SCZ-like behavior [22, 23]. Primers for qPCR were designed using the NCBI Primer Blast tool (Supplemental Tables S1 and S2). Primers were validated to have efficiency in the range of 90–110%. The CFX Opus 384 Real-Time PCR System (BioRad) with SensiFast SYBR (Meridian BioScience) was used to perform qPCR with the following protocol: an initial denaturation at 95°C for 3 min followed by 43 cycles of 95°C for 15 s, 58°C for 30 s, and 72°C for 30 s. Melt peaks for each primer were assessed to check for one distinct peak. Relative mRNA transcript was determined by normalizing each sample to the geometric mean of two housekeeping genes (β-actin and GAPDH) to determine $\Delta$Ct. The $\Delta$Ct of the controls were averaged, and this value was used to calculate the $\Delta \Delta$Ct of each sample. Relative gene expression was calculated using the equation 2^-ΔΔCt^. Genes that were found to be significant in the placenta of the rodent THC model were then measured in other rat tissues (PFC, Hipp) and human THC models (BeWo cells, adult rat brain, and organoid model) using either rat or human primers.

### Statistical analysis

Statistical analysis was performed using GraphPad Prism (Version 10.2.1). To determine the significance Student unpaired *t*-test, one-way ANOVA, and two-way ANOVA were performed where relevant. The ROUT method was used to determine outliers, and normality was calculated using the Shapiro–Wilk test. In cases where there were two unpaired groups and the data were not normal, a Mann–Whitney test was used. All data are shown with the mean $\pm$ SD unless stated otherwise. Results were deemed significant if $p<0.05$.

## Results

### Gestational exposure to edible THC in rats led to a decrease in fetal weight at GD19.5 with no significant change in maternal measures

There was no significant difference in maternal food intake, maternal weight gain, litter size, and live birth index between vehicle and THC groups (Table 2). Fetal measures were collected at GD19.5, and it was found that edible THC exposure significantly decreased the fetal weights and increased placenta: body weight ratio of offspring of both sexes (Table 3). Despite this significant increase, there was no significant change in placenta weight.

**Table 2:** Maternal measures of edible THC-exposed rat dams compared with controls

Maternal parameter	Vehicle (mean $\pm$ SD)	THC (mean $\pm$ SD)
Average maternal food intake GD 12–14 (g)	21.0 $\pm$ 1.48	19.4 $\pm$ 2.54
Average maternal food intake GD 15–18 (g)	20.8 $\pm$ 1.03	19.5 $\pm$ 1.86
Average maternal weight gain GD 7–18 (g)	62.3 $\pm$ 4.68	56.9 $\pm$ 7.98
Litter size (pups)	10.6 $\pm$ 2.20	12.7 $\pm$ 2.40
Live birth index (%)	89.1 $\pm$ 6.33	94.5 $\pm$ 6.33

Maternal measures were recorded every day starting on GD5 for both control and treatment groups. No significant differences between the control and treatment groups were found according to the Student unpaired *t*-test ($p<0.05$).

**Table 3:** Fetal measures of edible THC-exposed rat dams compared with controls

Fetal parameter	Vehicle (mean $\pm$ SD)	THC (mean $\pm$ SD)
*Fetal weight (g)* ^**^	1.67 $\pm$ 0.34	1.45 $\pm$ 0.15
Female weight (g)	1.63 $\pm$ 0.32	1.36 $\pm$ 0.19
Male weight (g)	1.69 $\pm$ 0.37	1.54 $\pm$ 0.11
*Placental weight (g)*	0.48 $\pm$ 0.08	0.45 $\pm$ 0.05
Female placenta weight (g)	0.47 $\pm$ 0.07	0.43 $\pm$ 0.06
Male placenta weight (g)	0.46 $\pm$ 0.04	0.47 $\pm$ 0.06
*Placenta: Body weight ratio* ^*^	0.29 $\pm$ 0.05	0.31 $\pm$ 0.03
Female P:B ratio	0.30 $\pm$ 0.06	0.32 $\pm$ 0.04
Male P:B ratio	0.28 $\pm$ 0.07	0.30 $\pm$ 0.03

Fetal measures were taken on GD 19.5. The vehicle fetal weight and placental weight were determined to be non-normal via the Shapiro–Wilk test and a Mann–Whitney test was used to determine significance. The placenta: body weight ratio was considered normal according to the Shapiro–Wilk test and a Student unpaired *t*-test was used to determine significance (^*^$p<0.05$, ^**^$p<0.01$).

### Seven prioritized placental genes associated with SCZ were differentially expressed in rodent placentae exposed to THC

**Figure 1:**
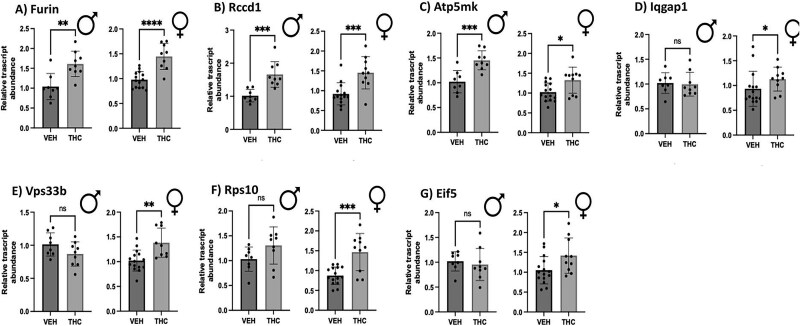
Relative transcript abundance of prioritized SCZ-associated genes altered in rodent placenta at GD19.5 after gestational-THC exposure. Steady-state mRNA expression of 20 targets was investigated using qPCR and displayed in the figure as mean ± SD of ($n=8\hbox{--} 15$/treatment/sex). Significance was determined using the Student unpaired *t*-tests with the exception of Iqgap1, which used a Mann–Whitney test as the data was considered non-normal according to the Shapiro–Wilk test (^*^$p<0.05$, ^**^$p<0.01$, ^***^$p<0.001$, and ^****^$p<0.0001$).

Based on previous publications [35, 36], 20 of the top differentially expressed genes associated with both SCZ and FGR were selected to be tested via qPCR in THC and vehicle-exposed placentae. Of the 20 placental genes tested that were associated with both FGR and SCZ risk, 13 revealed no significant difference after edible THC exposure during pregnancy (Supplemental Figure S1). However, four of the 20 genes were found to have significantly increased the relative mRNA transcript abundance exclusively in female offspring (Figure 1): *Iqgap1* (cell adhesion) [59], *Vps33b* (vesicle-mediated protein sorting) [60], *Rps10* (protein synthesis) [61], and *Eif5* (protein synthesis) [62]. An additional three genes were shown to have significantly increased steady-state mRNA expression in both male and female placentae (Figure 1). These three genes of interest were *Furin* (protein-cleaving enzyme) [63], *Rccd1* (hypothesized to be involved with chromatin reorganization) [64], and *Atp5mk* (involved in ATP synthesis) [65]. In summary, a total of seven of the 20 placental genes did show significant changes in mRNA levels in the placenta after prenatal THC exposure (Table 4).

**Table 4:** Prioritized genes found in the placenta where differential expression is linked to both increased SCZ risk and FGR [58, 59]

Prioritized placental schizophrenic genes found in FGR pregnancies		
THC-altered placental genes	Non-THC-altered placental genes	
*ATP5MK*	*ARHGAP1*	*NT5C2*
*EIF5*	*ARPC3*	*PAPPA2*
*FURIN*	*ATP242*	*PHF5A*
*IQGAP1*	*CLK1*	*SNX3*
*RCCD1*	*FES*	*TRIM8*
*RPS10*	*GID4*	*WBP1L*
*VPS33B*	*MSI2*	

Genes indicated on the left hand of the table had shown significant differences in relative transcript abundance in the placenta after chronic prenatal THC exposure using an edible THC rodent model. Genes indicated on the right hand of the table were tested in rodent THC-exposed placenta but showed no significant change.

### A subset of placental genes found to be differentially expressed in rodent model exhibited differential expression in the human BeWo cells treated with THC

**Figure 2:**
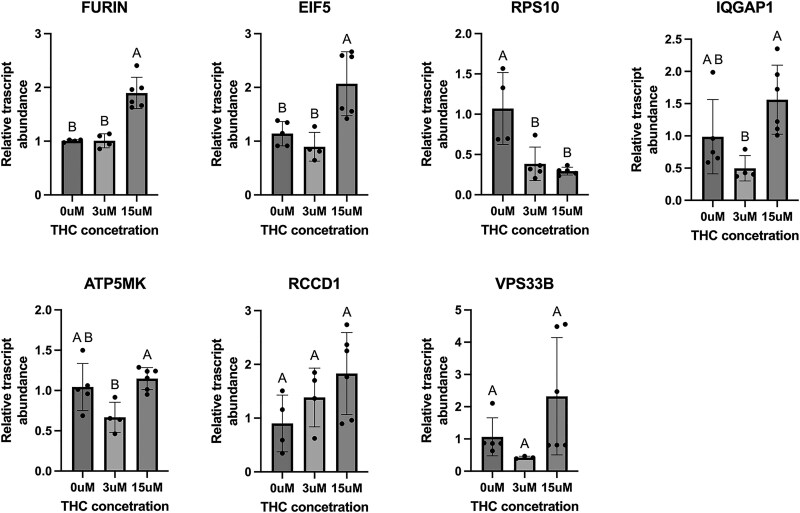
Significant genes altered in THC-exposed rodent placenta model are also differentially expressed in human placental BeWo cells exposed to THC for 24 h. BeWo cells were cultured in 0–15 μM for 24 h. Steady-state mRNA expression is displayed in the figure as mean ± SD ($n=4\hbox{--} 6$/treatment). Significance was determined with a two-tailed one-way ANOVA, and letters indicate significant differences between treatments ($p<0.05$).

Of the seven targets that showed a significant change in the THC-exposed rat placentae, three demonstrated significant increases in the BeWo cells after THC exposure (Figure 2) including *FURIN* (protein-cleaving enzyme), *IQGAP1* (cell adhesion), and EIF5 (protein synthesis), all in alignment with the results from the in vivo THC-exposed rat placentae. However, *RPS10* (protein synthesis) exhibited decreased relative expression, which is contrary to what was observed in the chronic prenatal THC-exposure rodent model.

### Gestational THC-exposed adult rodent PFC and hippocampus show some differential gene expression patterns similar to THC-exposed placenta concurrent with decreased %PPI

**Figure 3:**
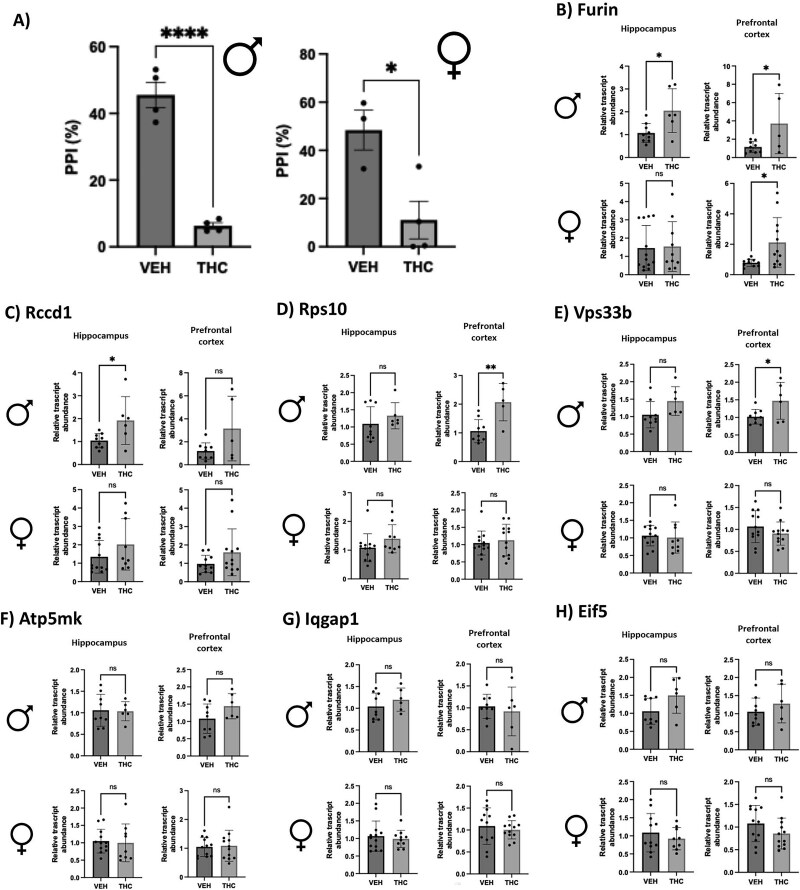
Adult rat PPI differences between gestational THC and vehicle-exposed offspring are concurrent with differential gene expression associated with SCZ in the PFC and Hipp. (A) Male and female adult rats (PND70) that were exposed to prenatal THC showed a significantly decreased PPI when compared with vehicle-exposed animals at the same time point. Mean ± SD of PPI% are plotted ($n=3\hbox{--} 5$/treatment/sex). Significance was determined using the Student unpaired *t*-tests (^*^$p<0.05$, ^****^$p<0.0001$). (B–E) The seven targets that showed significant change in the previous placenta rat model were investigated using qPCR in the adult rat PFC and Hipp. Mean ± SD of steady-state mRNA expression in PFC and Hipp is displayed in the figure ($n=5\hbox{--} 12$/treatment/sex) Significance was determined using the Student unpaired *t*-tests (^*^$p<0.05$).

In adulthood (PND70), male and female THC-exposed rat offspring demonstrated a significant decrease in %PPI (Figure 3A). To investigate if the THC-induced placental signature of SCZ-markers was also altered in the brain, we measured their expression in the PFC and Hipp at the same age (PND70). Of the genes tested, four were demonstrated to be significantly altered in at least one adult brain region after prenatal THC exposure (Figure 3B–E). Specifically, the relative transcript mRNA abundance of *Furin, Rps10*, and *Vps33b* was significantly increased in the adult male PFC of THC-exposed offspring. In the male Hipp, the expression of *Furin* and *Rccd1* mRNA was significantly increased after gestational THC exposure. The female PFC did show a significant increase in *Furin* transcript abundance after prenatal THC exposure.

### THC-exposed patient-derived human cerebral organoids show differential gene expression of targets altered in THC-exposed placenta

**Figure 4:**
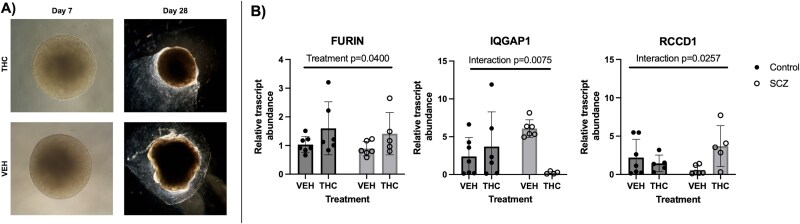
Relative transcript abundance of some significant genes found in the rodent placenta model was also altered in patient-derived control and SCZ cerebral organoids treated with THC. Images of the organoids derived from patient control and SCZ cell lines were taken throughout the differentiation process. Treatment of THC started on day 3 and continued until day 30. (A) Representative images of organoids on day 7 and day 28 show little difference between treatment groups. (B) Of the seven genes that showed significant change in the placenta, three showed significant changes in the organoid model. Mean ± SD of steady-state mRNA expression of Furin, Rccd1, and Iqgap1 in the organoids are shown ($n=5\hbox{--} 6$/treatment/cell line). Significance was determined using a two-tailed, two-way ANOVA (^*^$p<0.05$).

Since the rodent model investigated in this study showed overlap in altered gene expression in the placenta and adult brain, human cerebral brain organoids were generated and treated with THC during a development-equivalent phase to investigate if this signature of genes was also altered in the human-derived brain organoid model. Of all seven genes that were found to be differently expressed in rat placenta after THC exposure, *FURIN* (protein-cleaving enzyme) was the only gene to have significant differential expression in the cerebral organoid model between vehicle and THC exposure from both control and SCZ model organoids. Specifically, *FURIN* was found to have increased expression in both control and SCZ-derived organoids after treatment with THC from days 3 to 30 (Figure 4). *IQGAP1* (cell adhesion) and *RCCD1* (chromatin reorganization) had significantly different interactions between treatment and organoid type. This means that the expression of *IQGAP1* and *RCCD1* was dependent on the phenotype of cell lines. *IQGAP1* decreased after THC exposure in the SCZ organoids but increased in the control group after THC exposure. Conversely, *RCCD1* mRNA abundance was increased in the SCZ group after THC exposure and showed a slight decrease in the control group when treated with THC. The other four targets tested showed no significant changes in the expression between vehicle and THC exposure in control or SCZ organoids (Supplemental Figure S2).

## Discussion

As the use of cannabis in pregnancy increases, it is imperative to investigate any possible adverse neurobehavioral and/or metabolic outcomes that may persist in postnatal life as a result of prenatal exposure. Previous research indicates that early exposure to THC can lead to placental insufficiency and FGR [7, 10], which could be a risk factor for SCZ [8, 9, 30]. Elegant studies by Ursini et al. [35, 36] have found a connection between placental genes associated with SCZ specifically in FGR pregnancies, further implicating the role of the placenta–brain axis whereby poor placental development can negatively impact the proper trajectory of brain development. The current study indicates that prenatal THC exposure decreases fetal weight, which is indicative of growth restriction. Moreover, four prioritized established placental markers of SCZ associated with FGR (*FURIN, EIF5, RPS10,* and *IQGAP1*) were altered in the THC-exposed placenta in both rodent and human models, suggesting an increase in SCZ risk. Furthermore, this study revealed that there may be some overlap in differential gene expression between the placenta and the postnatal brain after gestational THC exposure with some sex-specific transcript changes.

Using our preclinical rodent model, we demonstrated that rat dams exposed to oral THC during pregnancy exhibited significantly decreased fetal weights at GD19.5 and increased placenta: body weight ratios when compared with vehicle-exposed animals, which is similar to the human outcomes observed after prenatal cannabis use [7]. The decrease in fetal weight aligns with previous preclinical research using *i.p.* injections [10, 11, 23] but may be distinct from what is seen in the preclinical vaping model [66]. Currently, the rodent THC inhalation model has shown conflicting results, with some studies reporting no weight differences between prenatal vapor-THC exposure and control rats [66], while other studies have demonstrated a decrease in birth weight after prenatal THC inhalation [67]. Despite this change in the fetal weight and placenta: body weight ratio, there were no significant changes in placental weight. Previously, studies have shown that prenatal THC (*i.p*) can lead to a larger placenta and smaller pups [10, 68], suggesting FGR. However, in the present study, the altered weight ratio still indicates that the THC-exposed placentae are supporting less growth, implying placental insufficiency.

Quantitative RT-PCR analyses identified differential expression of SCZ-associated genes in THC-exposed rodent placentae, consistent with prior findings in human placentae associated with FGR pregnancies and SCZ risk [35, 36]. Among the 20 prioritized genes derived from these studies, seven displayed significant differential expression: *Furin* (protein-cleaving) [69, 70], *Rccd1* (chromatin reorganization) [71, 72], *Atp5mk* (involved in ATP synthesis) [65], *Iqgap1* (cell adhesion) [73], *Vps33b* (vesicle-mediated protein sorting) [74], *Rps10* (protein synthesis) [75], and *Eif5* (protein synthesis) [62].

**Figure 5:**
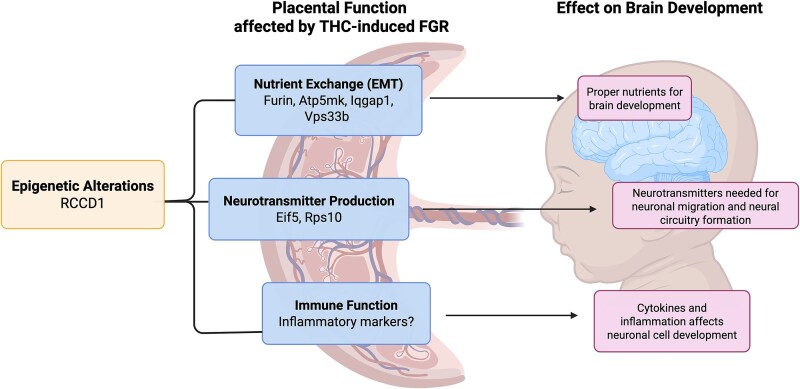
Summary of placenta–brain axis gene pathways implicated by prenatal THC exposure leading to FGR. The seven targets found to show significant changes in transcript abundance in the placenta after THC exposure are related to functions associated with proper placental function and growth which can impact brain development and lead to poor neuropsychiatric outcomes. This figure was created in BioRender.

While functionally different, five of the seven genes (*Furin, Rccd1, Atp5mk, Iqgap1,* and *Vps33b*) play an important role in epithelial-to-mesenchymal transition (EMT) [65, 69, 70, 72–74, 76]. EMT plays an important role during the development of the placenta, especially during the invasion of the trophoblast cells into the decidua and the remodeling of the spiral arteries [77, 78]. Additionally, the significant up-regulation of these protein synthesis-related genes (*Rps10* and *Eif5*) exclusively in the female placenta suggests sex-specific effects on protein metabolism [27] following THC exposure. Further studies are warranted to examine if these sex-specific effects on protein metabolism impact the placenta–brain axis [32]. Collectively, disruption in the expression of these genes can negatively impact placental function and result in FGR, which can have long-term effects on neurocognitive outcomes [8, 9] (Figure 5).

In comparison to previous studies, four out of the seven genes found to have significant changes in the expression after THC exposure (Furin, Iqgap1, Vps33b, and Rps10) in the rodent were also increased in the expression in human placentae associated with both FGR and SCZ [36]. In contrast, three genes (Atp5mk, Rccd1, and Eif5) exhibited decreased expression in human placentae associated with both FGR and SCZ, even though they were increased in the rodent THC-exposed rat placentae. Future studies are warranted to examine how overexpression of these three genes may impact cognitive outcomes and/or placental function. While rodent and human placentae share similarities (i.e.*,* hemochorial structure) [79], they exhibit some fundamental differences [80]. To evaluate translational relevance, we treated BeWo cells with THC, leading to significant alterations in the expression of four genes (*FURIN, EIF5, RPS10,* and *IQGAP1*) previously altered in the THC-exposed rodent placentae. This lends further support that THC can alter the expression of these genes associated with SCZ in the human placenta [35, 36]. Furthermore, treatment with THC led to decreased *RPS10* mRNA which is reciprocal of what was observed in the rodent model. The variations observed in expression patterns between these two models could be attributed to the difference in exposure time to THC or the heterogeneity of cell types seen in the rodent placenta models that were not present in the BeWo model. Despite these differences, these results indicate that THC exposure in either the rat or human placenta can alter genes that are associated with SCZ, supporting the hypothesis that gestational THC exposure can increase the risk of SCZ in offspring.

Interestingly, recent studies in pregnant non-human primates have also found that prenatal THC negatively impacts placental function [81] and can alter DNA methylation in genes associated with autism [82] that are related to the SCZ-associated genes found to be differentially expressed in the rodent and human placenta models in this study (i.e., IQGAP1 and VPS33B). The findings in the non-human primate model demonstrate the effect of THC on the placenta and the overlap of effects between several species’ models. Furthermore, these studies further imply that the risk of SCZ and other neurological disorders (i.e., autism) may originate in utero.

To follow up with changes in the identified THC-altered placental markers associated with SCZ, long-term neurobehavioral outcomes and brain gene expression patterns following prenatal THC exposure were investigated using PPI testing in adult rat offspring. This behavioral testing is of particular clinical interest as this test can be performed in humans, and those with SCZ exhibit a decrease in %PPI [25]. After PPI testing, both male and female adult offspring exposed to prenatal THC exhibited a decrease in %PPI. These findings align with what has been seen in the prenatal *i.p.* THC injection model [22] and clinical studies that show an increase in onset of psychotic-like behavior at a similar age equivalent (young adulthood) [20].

The THC-induced decrease in PPI combined with both placental insufficiency and altered placental expression in this study further establishes the role of the placenta–brain axis with regard to disturbances in the placenta, potentially acting as a predictor for developing neurodevelopmental disorders later in life. When we examined the placental-SCZ genes in the postnatal Hipp and PFC at PND70, notably four genes (*Furin, Rccd1, Rps10,* and *Vps33b*) exhibited increased steady-state mRNA levels in THC-exposed rat offspring concurrent with the observed decrease in PPI. The increase in the expression of these four genes aligns with what was previously observed in the THC-exposed placentae. Developmentally, this suggests that THC can elicit similar effects in the placenta and brain that could lead to an increased risk of developing SCZ.

To investigate if the genes altered in the rodent brain were also changed in a human brain model, patient-derived cerebral organoids from SCZ or control cell lines were treated with THC during a period of time (day 3–30) that would model early development (first trimester) [56]. Once again, treatment with THC increased the relative transcript levels of *FURIN,* in both SCZ and control cell lines after THC exposure. In addition, levels of *IQGAP1* and *RCCD1* relative transcript abundance were differentially altered in response to THC, depending on whether they came from a SCZ cell line or a control cell line. This suggests that those who have a genetic risk of SCZ may be differently affected by prenatal THC exposure. Collectively, the results obtained from both the animal brain model and the human cerebral organoid model indicate that the differential gene expression associated with SCZ in the placenta overlaps with changes in gene expression in the postnatal brain. Further research is warranted to determine how alterations in the expression of these specific genes may influence brain function and how THC may mechanistically influence their expression and relation to SCZ-like outcomes in offspring.

Although the THC-induced genes identified in this study have been implicated in other neuronal models of SCZ, the neurobiological function of many of these genes and their specific contribution to SCZ pathophysiology remain poorly characterized. Among the identified genes, *FURIN* has been the most extensively characterized. FURIN is a protein-cleaving enzyme that is required to activate certain important neurotrophins (i.e., BDNF) [83] that have implications in SCZ. Thus, disruption to this enzyme can have poor effects on neuropsychiatric outcomes [84]. Despite accumulating evidence implicating *FURIN* dysregulation in SCZ, the precise molecular mechanisms by which altered *FURIN* expression contributes to SCZ pathogenesis and symptomology remain unclear [84–86].

This study presents several limitations. The first stems from the variability and limited sample size inherent to patient-derived organoid models. Due to samples being derived from a human population, substantial inter-sample genetic variability remains. Such heterogeneity inevitably contributes to variability in the organoid outcomes observed, thereby constraining the generalizability of our results. Additionally, the organoid results were limited in statistical power, therefore, preventing this work from analyzing sex as a variable in this experiment. Finally, it should be stated that the human organoids were exposed to a lower concentration of THC (100 nM) than the concentration of THC we have used in this study and previously in BeWo cells [50]. This was to account for the longer exposure time of THC in the organoid model. A lower dose was selected based on the previous literature [56] to maintain cell viability.

Future work may want to investigate placental markers of SCZ that are independent of FGR in different in vitro and ex vivo models after THC exposure, since this work focused on FGR-related transcription changes. Moreover, single-cell RNA-sequencing would facilitate investigating multiple SCZ-genes that may be independent of FGR while also providing details about the cell types most affected by THC. Additionally, future research should pursue the function of the genes that found to be altered in both the brain and placenta. Currently, some of these differentially expressed genes are not well understood, such as *RCCD1*. If the exact function of these genes can be characterized in both the placenta and brain, this may further uncover the precise mechanisms linking how gestational THC exposure contributes to the etiology of SCZ in postnatal life.

To investigate these markers in a clinical context, future studies using larger randomized control studies are warranted to investigate if these placental SCZ-associated gene expressions are found in placentae from human pregnancies after cannabis exposure. However, there may be several confounding variables, including socioeconomic status and poly-substance use (i.e., tobacco, alcohol [87, 88]). In addition, given some of the SCZ biomarkers identified (i.e., Vps33b, Eif5, Rps10, Furin, and Atp5mk) impact protein secretion, they could also be measured in umbilical cord blood as a clinical biomarker of SCZ risk.

In conclusion, this study demonstrated that prenatal THC exposure in rodents can lead to alteration of gene expression in established prioritized human markers of SCZ in the placentae, culminating in FGR offspring which exhibit poor neurobehavioral outcomes (e.g., decreased %PPI). Several of these genes were confirmed to be altered in human placental cells. Additionally, the rodent model demonstrated some sex-specific effects whereby mRNA transcript changes after prenatal THC were more male-specific in the adult brain and more female-specific in the placentae. This is of particular interest as SCZ is slightly more common in males, and they demonstrate more severe cognitive symptoms [89]. Moreover, in the THC-exposed (i) Hipp and PFC, and (ii) THC-treated human control and SCZ organoids, a subset of the placental markers was also altered, implicating these markers may play a role in the developmental origins of SCZ. Of the genes examined in this study, *FURIN* is of particular interest as all THC models led to an increase in the relative mRNA transcript abundance of this gene. This may serve as a promising clinical biomarker in the placenta to identify those at greatest risk of SCZ early in life.

## Supplementary Material

Supplemental_Figure_1_600_dpi_ioaf191

Supplemental_Figure_2_600_dpi_ioaf191

Supplemental_Table_1_ioaf191

Supplemental_Table_2_ioaf191

## Data Availability

The datasets generated during and/or analyzed during the current study are available from the corresponding author on reasonable request.

## References

[1] Corsi DJ, Hsu H, Weiss D, Fell DB, Walker M. Trends and correlates of cannabis use in pregnancy: a population-based study in Ontario, Canada from 2012 to 2017. Can J Public Health 2019; 110:76–84. 30387034 10.17269/s41997-018-0148-0 PMC6335373

[2] Young-Wolff KC, Chi FW, Lapham GT, Alexeeff SE, Does MB, Ansley D, Campbell CI. Changes in prenatal cannabis use among pregnant individuals from 2012 to 2022. Obstet Gynecol 2024; 144:e101–e104. 39208448 10.1097/AOG.0000000000005711 PMC11407770

[3] Mejia MC, Sacca L, Ferris AH, Hennekens CH, Kitsantas P. Trends and variations in admissions for cannabis use disorder among pregnant women in United States. J Perinat Med 2024;53:402–406. 10.1515/jpm-2024-0487 39716832

[4] Manning S, Drover A. Parental perceptions and patterns of cannabis use during pregnancy and breastfeeding at a Canadian tertiary obstetrics Centre. J Obstet Gynaecol Can 2020; 42:681.

[5] Muckle G, Laflamme D, Gagnon J, Boucher O, Jacobson JL, Jacobson SW. Alcohol, smoking and drug use among inuit women of childbearing age during pregnancy and the risk to children. Alcohol Clin Exp Res 2011; 35:1081–1091. 21332531 10.1111/j.1530-0277.2011.01441.x PMC3097283

[6] Gibson GT, Baghurst PA, Colley DP. Maternal alcohol, tobacco and cannabis consumption and the outcome of pregnancy. Aust N Z J Obstet Gynaecol 1983; 23:15–19. 6575752 10.1111/j.1479-828x.1983.tb00151.x

[7] Metz TD, Allshouse AA, McMillin GA, Greene T, Chung JH, Grobman WA, Haas DM, Mercer BM, Parry S, Reddy UM, Saade GR, Simhan HN, et al. Cannabis exposure and adverse pregnancy outcomes related to placental function. JAMA 2023; 330:2191–2199. 38085313 10.1001/jama.2023.21146 PMC10716715

[8] Cannon M, Jones PB, Murray RM. Obstetric complications and schizophrenia: historical and meta-analytic review. Am J Psychiatry 2002; 159:1080–1092. 12091183 10.1176/appi.ajp.159.7.1080

[9] Eide MG, Moster D, Irgens LM, Reichborn-Kjennerud T, Stoltenberg C, Skjærven R, Susser E, Abel K. Degree of fetal growth restriction associated with schizophrenia risk in a national cohort. Psychol Med 2013; 43:2057–2066. 23298736 10.1017/S003329171200267X

[10] Natale BV, Gustin KN, Lee K, Holloway AC, Laviolette SR, Natale DRC, Hardy DB. Δ9-tetrahydrocannabinol exposure during rat pregnancy leads to symmetrical fetal growth restriction and labyrinth-specific vascular defects in the placenta. Sci Rep 2020; 10:544. 31953475 10.1038/s41598-019-57318-6 PMC6969028

[11] Lee K, Laviolette SR, Hardy DB. Exposure to Δ9-tetrahydrocannabinol during rat pregnancy leads to impaired cardiac dysfunction in postnatal life. Pediatr Res 2021; 90:532–539. 33879850 10.1038/s41390-021-01511-9 PMC8519775

[12] Lu H-C, Mackie K. Review of the endocannabinoid system. Biol Psychiatry Cogn Neurosci Neuroimaging 2021; 6:607–615. 32980261 10.1016/j.bpsc.2020.07.016 PMC7855189

[13] Chayasirisobhon S . Mechanisms of action and pharmacokinetics of cannabis. Perm J 2020; 25:19.200. 10.7812/TPP/19.200 PMC8803256 33635755

[14] Maia J, Fonseca B, Teixeira N, Correia-da-Silva G. The fundamental role of the endocannabinoid system in endometrium and placenta: implications in pathophysiological aspects of uterine and pregnancy disorders. Hum Reprod Update 2020; 26:586–602. 32347309 10.1093/humupd/dmaa005 PMC7317288

[15] Andréasson S, Engström A, Allebeck P, Rydberg U. CANNABIS AND SCHIZOPHRENIA a longitudinal study of Swedish conscripts. The Lancet 1987; 330:1483–1486. 10.1016/s0140-6736(87)92620-1 2892048

[16] Arseneault L, Cannon M, Poulton R, Murray R, Caspi A, Moffitt TE. Cannabis use in adolescence and risk for adult psychosis: longitudinal prospective study. BMJ 2002; 325:1212–1213. 12446537 10.1136/bmj.325.7374.1212 PMC135493

[17] Weinberger DR . The neurodevelopmental origins of schizophrenia in the penumbra of genomic medicine. World Psychiatry 2017; 16:225–226. 28941096 10.1002/wps.20474 PMC5608828

[18] Pearson NT, Berry JH. Cannabis and psychosis through the lens of DSM-5. Int J Environ Res Public Health 2019; 16:4149. 31661851 10.3390/ijerph16214149 PMC6861931

[19] Paul SE, Hatoum AS, Fine JD, Johnson EC, Hansen I, Karcher NR, Moreau AL, Bondy E, Qu Y, Carter EB, Rogers CE, Agrawal A, et al. Associations between prenatal cannabis exposure and childhood outcomes: results from the ABCD study. JAMA Psychiatry 2021; 78:64–76. 32965490 10.1001/jamapsychiatry.2020.2902 PMC7512132

[20] Day NL, Goldschmidt L, Day R, Larkby C, Richardson GA. Prenatal marijuana exposure, age of marijuana initiation, and the development of psychotic symptoms in young adults. Psychol Med 2015; 45:1779–1787. 25534593 10.1017/S0033291714002906 PMC8128137

[21] Bolhuis K, Kushner SA, Yalniz S, Hillegers MHJ, Jaddoe VWV, Tiemeier H, Marroun HEI. Maternal and paternal cannabis use during pregnancy and the risk of psychotic-like experiences in the offspring. Schizophr Res 2018; 202:322–327. 29983267 10.1016/j.schres.2018.06.067

[22] Sarikahya MH, Cousineau SL, De Felice M, Szkudlarek HJ, Wong KKW, DeVuono MV, Lee K, Rodríguez-Ruiz M, Gummerson D, Proud E, Ng THJ, Hudson R, et al. Prenatal THC exposure induces long-term, sex-dependent cognitive dysfunction associated with lipidomic and neuronal pathology in the prefrontal cortex-hippocampal network. Mol Psychiatry 2023; 28:4234–4250. 10.1038/s41380-023-02190-0 37525013

[23] DeVuono MV, Nashed MG, Sarikahya MH, Kocsis A, Lee K, Vanin SR, Hudson R, Lonnee EP, Rushlow WJ, Hardy DB, Laviolette SR. Prenatal tetrahydrocannabinol and cannabidiol exposure produce sex-specific pathophysiological phenotypes in the adolescent prefrontal cortex and hippocampus. Neurobiol Dis 2024; 199:106588. 38960101 10.1016/j.nbd.2024.106588

[24] Sarikahya MH, Cousineau S, De Felice M, Lee K, Wong KK, DeVuono MV, Jung T, Rodríguez-Ruiz M, Ng THJ, Gummerson D, Proud E, Hardy DB, et al. Prenatal THC exposure induces sex-dependent neuropsychiatric endophenotypes in offspring and long-term disruptions in fatty-acid signaling pathways directly in the mesolimbic circuitry. eNeuro 2022; 9:ENEURO.0253-22.2022. 10.1523/ENEURO.0253-22.2022 PMC9557330 36171057

[25] Mena A, Ruiz-Salas JC, Puentes A, Dorado I, Ruiz-Veguilla M, De la Casa LG. Reduced prepulse inhibition as a biomarker of schizophrenia. Front Behav Neurosci 2016; 10:202. 27803654 10.3389/fnbeh.2016.00202 PMC5067522

[26] Raffard S, Lebrun C, Bayard S, Macgregor A, Capdevielle D. Self-awareness deficits of cognitive impairment in individuals with schizophrenia. Really? Front Psychiatry 2020; 11:731. 10.3389/fpsyt.2020.00731 PMC7406784 32848912

[27] Burton GJ, Jauniaux E. Pathophysiology of placental-derived fetal growth restriction. Am J Obstet Gynecol 2018; 218:S745–S761. 29422210 10.1016/j.ajog.2017.11.577

[28] Burkhardt T, Schäffer L, Schneider C, Zimmermann R, Kurmanavicius J. Reference values for the weight of freshly delivered term placentas and for placental weight–birth weight ratios. Eur J Obstet Gynecol Reprod Biol 2006; 128:248–252. 16377060 10.1016/j.ejogrb.2005.10.032

[29] Miller SL, Huppi PS, Mallard C. The consequences of fetal growth restriction on brain structure and neurodevelopmental outcome. J Physiol 2016; 594:807–823. 26607046 10.1113/JP271402 PMC4753264

[30] Nielsen PR, Mortensen PB, Dalman C, Henriksen TB, Pedersen MG, Pedersen CB, Agerbo E. Fetal growth and schizophrenia: a nested case-control and case-sibling study. Schizophr Bull 2013; 39:1337–1342. 23236080 10.1093/schbul/sbs148 PMC3796081

[31] Allgäuer L, Cabungcal J-H, Yzydorczyk C, Do KQ, Dwir D. Low protein-induced intrauterine growth restriction as a risk factor for schizophrenia phenotype in a rat model: assessing the role of oxidative stress and neuroinflammation interaction. Transl Psychiatry 2023; 13:30. 36720849 10.1038/s41398-023-02322-8 PMC9889339

[32] Rosenfeld CS . The placenta-brain-axis. J Neurosci Res 2021; 99:271–283. 32108381 10.1002/jnr.24603 PMC7483131

[33] Lester BM, Marsit CJ. Epigenetic mechanisms in the placenta related to infant neurodevelopment. Epigenomics 2018; 10:321–333. 29381081 10.2217/epi-2016-0171 PMC6219448

[34] Cilleros-Portet A, Lesseur C, Marí S, Cosin-Tomas M, Lozano M, Irizar A, Burt A, García-Santisteban I, Garrido-Martín D, Escaramís G, Hernangomez-Laderas A, Soler-Blasco R, et al. Potentially causal associations between placental DNA methylation and schizophrenia and other neuropsychiatric disorders. Nat Commun 2025; 16:2431. 40087310 10.1038/s41467-025-57760-3 PMC11909199

[35] Ursini G, Punzi G, Chen Q, Marenco S, Robinson JF, Porcelli A, Hamilton EG, Mitjans M, Maddalena G, Begemann M, Seidel J, Yanamori H, et al. Convergence of placenta biology and genetic risk for schizophrenia. Nat Med 2018; 24:792–801. 29808008 10.1038/s41591-018-0021-y

[36] Ursini G, Di Carlo P, Mukherjee S, Chen Q, Han S, Kim J, Deyssenroth M, Marsit CJ, Chen J, Hao K, Punzi G, Weinberger DR. Prioritization of potential causative genes for schizophrenia in placenta. Nat Commun 2023; 14:2613. 37188697 10.1038/s41467-023-38140-1 PMC10185564

[37] DiNieri JA, Hurd YL. Rat models of prenatal and adolescent cannabis exposure. In: Kobeissy FH (ed.), Psychiatric Disorders: Methods and Protocols. Totowa, NJ: Humana Press; 2012: 231–242. 10.1007/978-1-61779-458-2_14 22231817

[38] Bonnin A, de Miguel R, Hernández ML, Ramos JA, Fernández-Ruiz JJ. The prenatal exposure to *δ*9-tetrahydrocannabinol affects the gene expression and the activity of tyrosine hydroxylase during early brain development. Life Sci 1995; 56:2177–2184. 7776847 10.1016/0024-3205(95)00205-k

[39] Huestis MA, Cone EJ. Relationship of Δ9-tetrahydrocannabinol concentrations in oral fluid and plasma after controlled administration of smoked cannabis. J Anal Toxicol 2004; 28:394–399. 15516285 10.1093/jat/28.6.394

[40] Huestis MA, Henningfield JE, Cone EJ. Blood cannabinoids. II. Models for the prediction of time of marijuana exposure from plasma concentrations of Δ9-tetrahydrocannabinol (THC) and 11-nor-9-carboxy-Δ9-tetrahydrocannabinol (THCCOOH). J Anal Toxicol 1992; 16:283–290. 1338216 10.1093/jat/16.5.283

[41] Hunault CC, Mensinga TT, de Vries I, Kelholt-Dijkman HH, Hoek J, Kruidenier M, Leenders MEC, Meulenbelt J. Delta-9-tetrahydrocannabinol (THC) serum concentrations and pharmacological effects in males after smoking a combination of tobacco and cannabis containing up to 69 mg THC. Psychopharmacology (Berl) 2008; 201:171–181. 18695931 10.1007/s00213-008-1260-2

[42] Lallai V, Manca L, Sherafat Y, Fowler CD. Effects of prenatal nicotine, THC, or co-exposure on cognitive behaviors in adolescent male and female rats. Nicotine Tob Res 2022; 24:1150–1160. 35090174 10.1093/ntr/ntac018 PMC9278841

[43] Lee K, Vanin S, Nashed M, Sarikahya MH, Laviolette SR, Natale DRC, Hardy DB. Cannabidiol exposure during gestation leads to adverse cardiac outcomes early in postnatal life in male rat offspring. Cannabis Cannabinoid Res 2024; 9:781–796. 38358335 10.1089/can.2023.0213

[44] Renard J, Rosen LG, Loureiro M, De Oliveira C, Schmid S, Rushlow WJ, Laviolette SR. Adolescent cannabinoid exposure induces a persistent sub-cortical hyper-dopaminergic state and associated molecular adaptations in the prefrontal cortex. Cereb Cortex 2017; 27:1297–1310. 26733534 10.1093/cercor/bhv335

[45] Loranger AW . Sex difference in age at onset of schizophrenia. Arch Gen Psychiatry 1984; 41:157–161. 6696597 10.1001/archpsyc.1984.01790130053007

[46] Zhan N, Sham PC, So H-C, Lui SSY. The genetic basis of onset age in schizophrenia: evidence and models. Front Genet 2023; 14:1163361. 37441552 10.3389/fgene.2023.1163361 PMC10333597

[47] Imbarak N, Abdel-Aziz HI, Farghaly LM, Hosny S. Effect of mesenchymal stem cells versus aloe vera on healing of deep second-degree burn. Stem Cell Investig 2021; 8:12. 10.21037/sci-2020-030 PMC8256119 34268441

[48] Khare M, Taylor AH, Konje JC, Bell SC. Δ9-Tetrahydrocannabinol inhibits cytotrophoblast cell proliferation and modulates gene transcription. Mol Hum Reprod 2006; 12:321–333. 16597638 10.1093/molehr/gal036

[49] Walker OS, Ragos R, Gurm H, Lapierre M, May LL, Raha S. Delta-9-tetrahydrocannabinol disrupts mitochondrial function and attenuates syncytialization in human placental BeWo cells. Physiol Rep 2020; 8:e14476. 10.14814/phy2.14476 PMC7336740 32628362

[50] Lojpur T, Easton Z, Raez-Villanueva S, Laviolette S, Holloway AC, Hardy DB. Δ9-Tetrahydrocannabinol leads to endoplasmic reticulum stress and mitochondrial dysfunction in human BeWo trophoblasts. Reprod Toxicol Elmsford N 2019; 87:21–31. 10.1016/j.reprotox.2019.04.008 31054322

[51] Chang X, Bian Y, He Q, Yao J, Zhu J, Wu J, Wang K, Duan T. Suppression of STAT3 signaling by Δ9-tetrahydrocannabinol (THC) induces trophoblast dysfunction. Cell Physiol Biochem 2017; 42:537–550. 28578322 10.1159/000477603

[52] Proud EK, Rodríguez-Ruiz M, Gummerson DM, Vanin S, Hardy DB, Rushlow WJ, Laviolette SR. Chronic nicotine exposure induces molecular and transcriptomic endophenotypes associated with mood and anxiety disorders in a cerebral organoid neurodevelopmental model. Front Pharmacol 2024; 15:1473213. 39764466 10.3389/fphar.2024.1473213 PMC11701148

[53] Watanabe M, Buth JE, Haney JR, Vishlaghi N, Turcios F, Elahi LS, Gu W, Pearson CA, Kurdian A, Baliaouri NV, Collier AJ, Miranda OA, et al. TGFβ superfamily signaling regulates the state of human stem cell pluripotency and capacity to create well-structured telencephalic organoids. Stem Cell Rep 2022; 17:2220–2238. 10.1016/j.stemcr.2022.08.013 PMC9561534 36179695

[54] Lancaster MA, Knoblich JA. Generation of cerebral organoids from human pluripotent stem cells. Nat Protoc 2014; 9:2329–2340. 25188634 10.1038/nprot.2014.158 PMC4160653

[55] Lancaster MA, Corsini NS, Wolfinger S, Gustafson EH, Phillips AW, Burkard TR, Otani T, Livesey FJ, Knoblich JA. Guided self-organization and cortical plate formation in human brain organoids. Nat Biotechnol 2017; 35:659–666. 28562594 10.1038/nbt.3906 PMC5824977

[56] Ao Z, Cai H, Havert DJ, Wu Z, Gong Z, Beggs JM, Mackie K, Guo F. One-stop microfluidic assembly of human brain organoids to model prenatal cannabis exposure. Anal Chem 2020; 92:4630–4638. 32070103 10.1021/acs.analchem.0c00205

[57] Wible CG, Anderson J, Shenton ME, Kricun A, Hirayasu Y, Tanaka S, Levitt JJ, O’Donnell BF, Kikinis R, Jolesz FA, McCarley RW. Prefrontal cortex, negative symptoms, and schizophrenia: an MRI study. Psychiatry Res Neuroimaging 2001; 108:65–78. 10.1016/s0925-4927(01)00109-3 PMC2845854 11738541

[58] Lieberman JA, Girgis RR, Brucato G, Moore H, Provenzano F, Kegeles L, Javitt D, Kantrowitz J, Wall MM, Corcoran CM, Schobel SA, Small SA. Hippocampal dysfunction in the pathophysiology of schizophrenia: a selective review and hypothesis for early detection and intervention. Mol Psychiatry 2018; 23:1764–1772. 29311665 10.1038/mp.2017.249 PMC6037569

[59] Abel AM, Schuldt KM, Rajasekaran K, Hwang D, Riese MJ, Rao S, Thakar MS, Malarkannan S. IQGAP1: insights into the function of a molecular puppeteer. Mol Immunol 2015; 65:336–349. 25733387 10.1016/j.molimm.2015.02.012 PMC4480615

[60] Liu RJY, Al-Molieh Y, Chen SZ, Drobac M, Urban D, Chen CH, Yao HHY, Geng RSQ, Li L, Pluthero FG, Benlekbir S, Rubinstein JL, et al. The Sec1-Munc18 protein VPS33B forms a uniquely bidirectional complex with VPS16B. J Biol Chem 2023; 299:104718. 37062417 10.1016/j.jbc.2023.104718 PMC10208892

[61] Warner JR, McIntosh KB. How common are extraribosomal functions of ribosomal proteins? Mol Cell 2009; 34:3–11. 19362532 10.1016/j.molcel.2009.03.006 PMC2679180

[62] Gotoh S, Mori K, Fujino Y, Kawabe Y, Yamashita T, Omi T, Nagata K, Tagami S, Nagai Y, Ikeda M. eIF5 stimulates the CUG initiation of RAN translation of poly-GA dipeptide repeat protein (DPR) in *C9orf72* FTLD/ALS. J Biol Chem 2024; 300:105703. 38301895 10.1016/j.jbc.2024.105703 PMC10904283

[63] Than ME, Huber R, Henrich S, Bartunik HD, Bourenkov GP, Bode W. The endoproteinase furin contains two essential Ca2+ ions stabilizing its N-terminus and the unique S1 specificity pocket. Acta Crystallogr Sect D 2005; 61:505–512. 15858259 10.1107/S0907444905002556

[64] Marcon E, Ni Z, Pu S, Turinsky AL, Trimble SS, Olsen JB, Silverman-Gavrila R, Silverman-Gavrila L, Phanse S, Guo H, Zhong G, Guo X, et al. Human-chromatin-related protein interactions identify a demethylase complex required for chromosome segregation. Cell Rep 2014; 8:297–310. 24981860 10.1016/j.celrep.2014.05.050

[65] Kontro H, Cannino G, Rustin P, Dufour E, Kainulainen H. DAPIT over-expression modulates glucose metabolism and cell behaviour in HEK293T cells. PLoS One 2015; 10:e0131990. 26161955 10.1371/journal.pone.0131990 PMC4498893

[66] Breit KR, Rodriguez CG, Hussain S, Thomas KJ, Zeigler M, Gerasimidis I, Thomas JD. A model of combined exposure to nicotine and tetrahydrocannabinol via electronic cigarettes in pregnant rats. Front Neurosci 2022; 16:866722. 35368251 10.3389/fnins.2022.866722 PMC8966542

[67] Roeder NM, Penman SL, Richardson BJ, Wang J, Freeman-Striegel L, Khan A, Pareek O, Weiss M, Mohr P, Eiden RD, Chakraborty S, Thanos PK. Vaporized Δ9-THC in utero results in reduced birthweight, increased locomotion, and altered wake-cycle activity dependent on dose, sex, and diet in the offspring. Life Sci 2024; 340:122447. 38246518 10.1016/j.lfs.2024.122447

[68] Benevenuto SG, Domenico MD, Martins MAG, Costa NS, de Souza ARL, Costa JL, Tavares MFM, Dolhnikoff M, Veras MM. Recreational use of marijuana during pregnancy and negative gestational and fetal outcomes: an experimental study in mice. Toxicology 2017; 376:94–101. 27234314 10.1016/j.tox.2016.05.020

[69] Zhou Z, Shen T, Zhang B-H, Lv X-Y, Lin H-Y, Zhu C, Xue L-Q, Wang H. The proprotein convertase furin in human trophoblast: possible role in promoting trophoblast cell migration and invasion. Placenta 2009; 30:929–938. 19853298 10.1016/j.placenta.2009.09.003

[70] Morosin SK, Delforce SJ, Corbisier de Meaultsart C, Lumbers ER, Pringle KG. FURIN and placental syncytialisation: a cautionary tale. Cell Death Dis 2021; 12:635. 34155192 10.1038/s41419-021-03898-z PMC8217546

[71] Wu J, He Z, Yang X-M, Li K-L, Wang D-L, Sun F-L. RCCD1 depletion attenuates TGF-β-induced EMT and cell migration by stabilizing cytoskeletal microtubules in NSCLC cells. Cancer Lett 2017; 400:18–29. 28455245 10.1016/j.canlet.2017.04.021

[72] Han S, Lin M, Wu L, Lin X, Chen M, Hu C, Bao A, Fang Z, Zhu F. E2F1 modulates RCCD1 expression to participate in the initiation and progression of EMT in colorectal cancer. Pathol - Res Pract 2024; 260:155429. 39024731 10.1016/j.prp.2024.155429

[73] Hu W, Wang Z, Zhang S, Lu X, Wu J, Yu K, Ji A, Lu W, Wang Z, Wu J, Jiang C. IQGAP1 promotes pancreatic cancer progression and epithelial-mesenchymal transition (EMT) through Wnt/β-catenin signaling. Sci Rep 2019; 9:7539. 31101875 10.1038/s41598-019-44048-y PMC6525164

[74] Liu Z, Liu J, Li Y, Wang H, Liang Z, Deng X, Fu Q, Fang W, Xu P. VPS33B suppresses lung adenocarcinoma metastasis and chemoresistance to cisplatin. Genes Dis 2020; 8:307–319. 33997178 10.1016/j.gendis.2019.12.009 PMC8093570

[75] Lee SJ, Swanson MJ, Sattlegger E. Gcn1 contacts the small ribosomal protein Rps10, which is required for full activation of the protein kinase Gcn2. Biochem J 2015; 466:547–559. 25437641 10.1042/BJ20140782

[76] Wu J, He Z, Yang X-M, Li K-L, Wang D-L, Sun F-L. RCCD1 depletion attenuates TGF-β-induced EMT and cell migration by stabilizing cytoskeletal microtubules in NSCLC cells. Cancer Lett 2017; 400:18–29. 28455245 10.1016/j.canlet.2017.04.021

[77] Davies J, Pollheimer J, Yong HEJ, Kokkinos MI, Kalionis B, Knöfler M, Murthi P. Epithelial-mesenchymal transition during extravillous trophoblast differentiation. Cell Adh Migr 2016; 10:310–321. 27070187 10.1080/19336918.2016.1170258 PMC4951171

[78] Chen X, Tong C, Li H, Peng W, Li R, Luo X, Ge H, Ran Y, Li Q, Liu Y, Xiong X, Bai Y, et al. Dysregulated expression of RPS4Y1 (ribosomal protein S4, Y-linked 1) impairs STAT3 (signal transducer and activator of transcription 3) signaling to suppress trophoblast cell migration and invasion in preeclampsia. Hypertension 2018; 71:481–490. 29378854 10.1161/HYPERTENSIONAHA.117.10250

[79] Soares MJ, Chakraborty D, Karim Rumi MA, Konno T, Renaud SJ. Rat placentation: an experimental model for investigating the hemochorial maternal-fetal interface. Placenta 2012; 33:233–243. 22284666 10.1016/j.placenta.2011.11.026 PMC3288880

[80] Aguilera N, Salas-Pérez F, Ortíz M, Álvarez D, Echiburú B, Maliqueo M. Rodent models in placental research. Implications for fetal origins of adult disease. Anim Reprod 2022; 19:e20210134. 35493783 10.1590/1984-3143-AR2021-0134 PMC9037606

[81] Roberts VHJ, Schabel MC, Boniface ER, D’Mello RJ, Morgan TK, Terrobias JJD, Graham JA, Borgelt LM, Grant KA, Sullivan EL, Lo JO. Chronic prenatal delta-9-tetrahydrocannabinol exposure adversely impacts placental function and development in a rhesus macaque model. Sci Rep 2022; 12:20260. 36424495 10.1038/s41598-022-24401-4 PMC9691736

[82] Shorey-Kendrick LE, Roberts VHJ, D’Mello RJ, Sullivan EL, Murphy SK, Mccarty OJT, Schust DJ, Hedges JC, Mitchell AJ, Terrobias JJD, Easley CA, Spindel ER, et al. Prenatal delta-9-tetrahydrocannabinol exposure is associated with changes in rhesus macaque DNA methylation enriched for autism genes. Clin Epigenetics 2023; 15:104. 37415206 10.1186/s13148-023-01519-4 PMC10324248

[83] Wang M, Xie Y, Qin D. Proteolytic cleavage of proBDNF to mBDNF in neuropsychiatric and neurodegenerative diseases. Brain Res Bull 2021; 166:172–184. 33202257 10.1016/j.brainresbull.2020.11.005

[84] Zhang Y, Gao X, Bai X, Yao S, Chang Y-Z, Gao G. The emerging role of furin in neurodegenerative and neuropsychiatric diseases. Transl Neurodegener 2022; 11:39. 35996194 10.1186/s40035-022-00313-1 PMC9395820

[85] Foka K, Georganta E-M, Semelidou O, Skoulakis EMC. Loss of the schizophrenia-linked furin protein from drosophila mushroom body neurons results in antipsychotic-reversible habituation deficits. J Neurosci 2022; 42:7496–7511. 36028314 10.1523/JNEUROSCI.1055-22.2022 PMC9525163

[86] Fromer M, Roussos P, Sieberts SK, Johnson JS, Kavanagh DH, Perumal TM, Ruderfer DM, Oh EC, Topol A, Shah HR, Klei LL, Kramer R, et al. Gene expression elucidates functional impact of polygenic risk for schizophrenia. Nat Neurosci 2016; 19:1442–1453. 27668389 10.1038/nn.4399 PMC5083142

[87] El Marroun H, Tiemeier H, Steegers EAP, Jaddoe VWV, Hofman A, Verhulst FC, van den Brink W, Huizink AC. Intrauterine cannabis exposure affects Fetal growth trajectories: the generation R study. J Am Acad Child Adolesc Psychiatry 2009; 48:1173–1181. 19858757 10.1097/CHI.0b013e3181bfa8ee

[88] Campbell EE, Gilliland J, Dworatzek PDN, Vrijer BD, Penava D, Seabrook JA. Socioeconomic status and adverse birth outcomes: a population-based Canadian sample. J Biosoc Sci 2018; 50:102–113. 28270256 10.1017/S0021932017000062

[89] Han M, Huang X-F, Chen DC, Xiu MH, Hui L, Liu H, Kosten TR, Zhang XY. Gender differences in cognitive function of patients with chronic schizophrenia. Prog Neuropsychopharmacol Biol Psychiatry 2012; 39:358–363. 22820676 10.1016/j.pnpbp.2012.07.010

