# Supplementary figures and images for: Identifying established human placental markers of schizophrenia in rodents after gestational ∆9-tetrahydrocannabinol exposure
Source: Biol Reprod. 2025 Aug 19;114(1):246–58. doi: 10.1093/biolre/ioaf191 (PMC12808552; doi:10.1093/biolre/ioaf191)

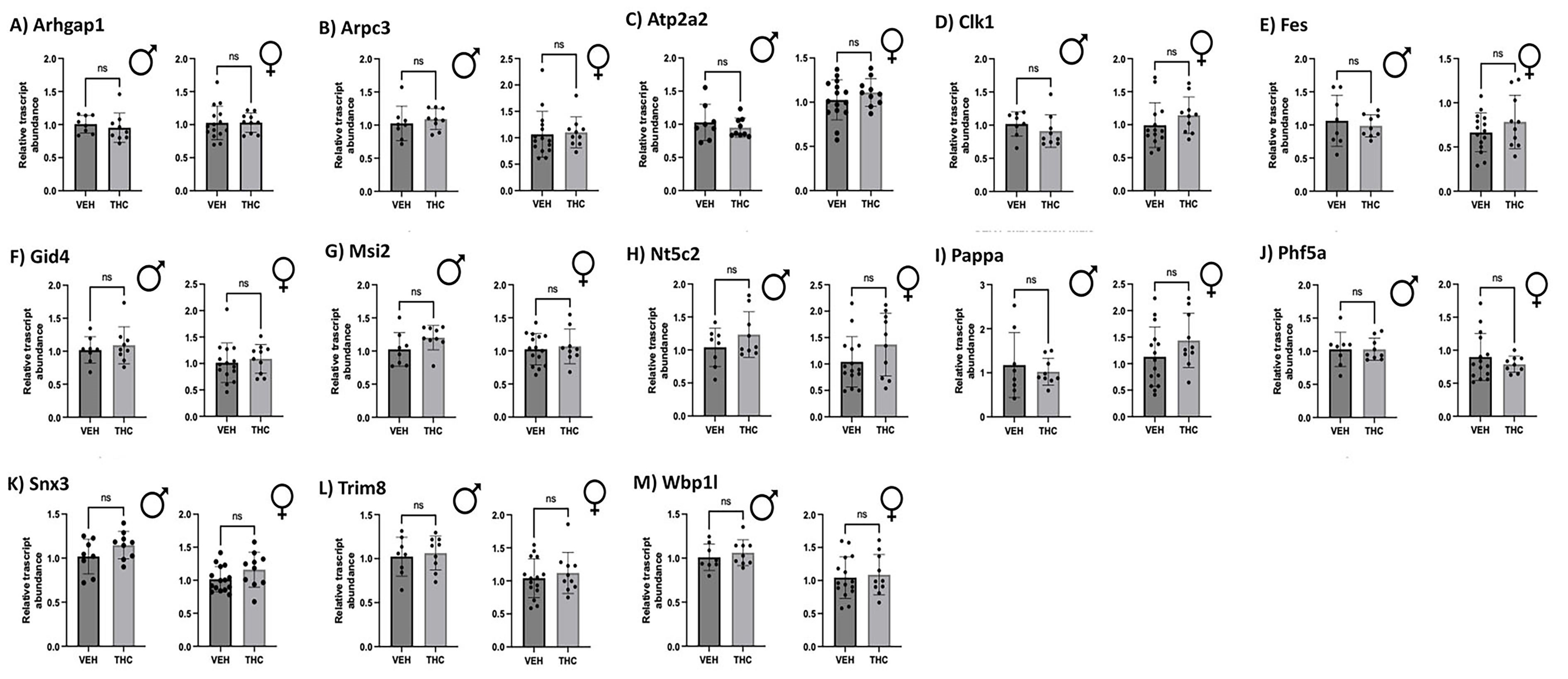

Supplement: Supplemental_Figure_1_600_dpi_ioaf191 [file supplemental_figure_1_600_dpi_ioaf191.jpeg]

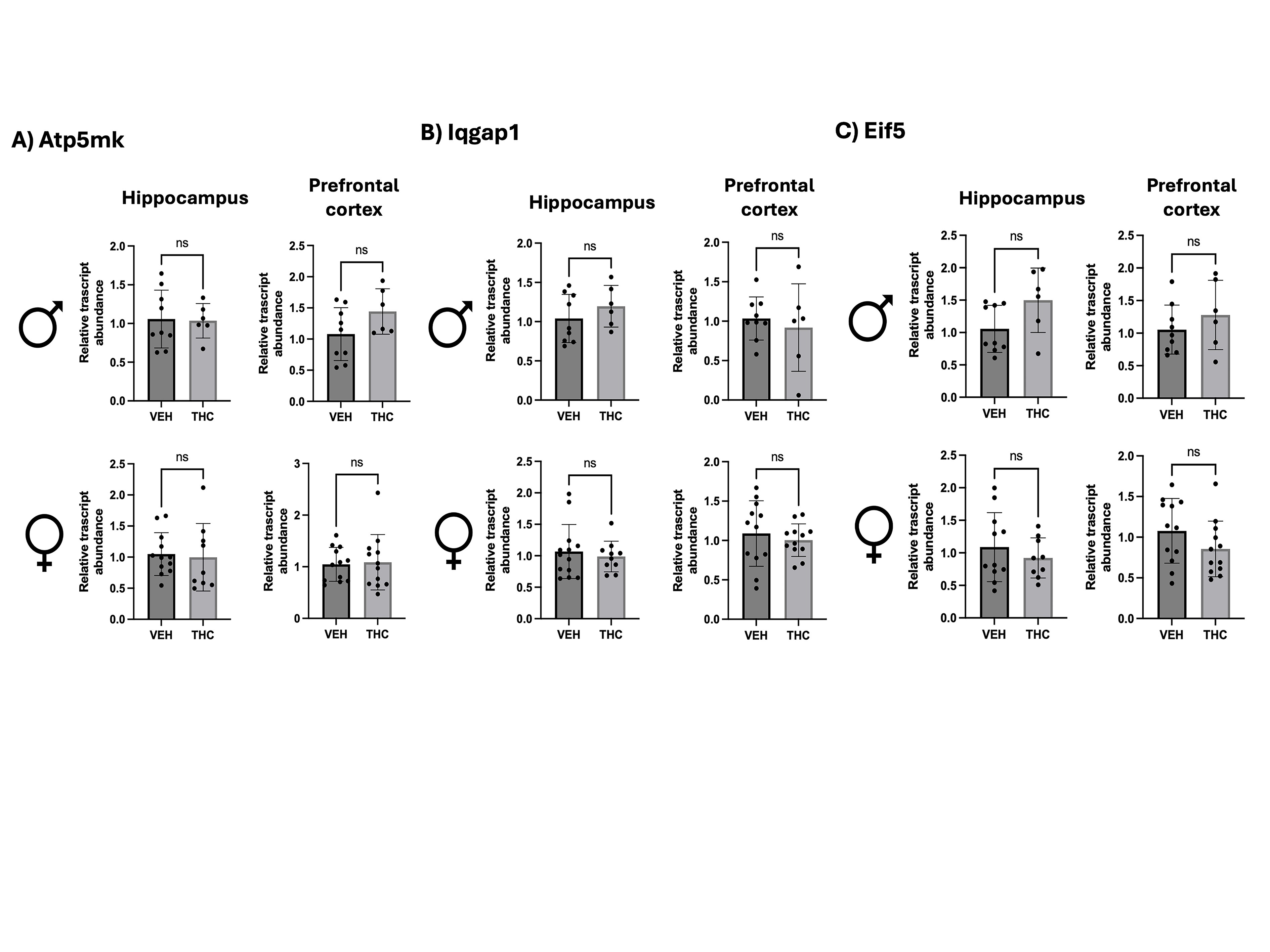

Supplement: Supplemental_Figure_2_600_dpi_ioaf191 [file supplemental_figure_2_600_dpi_ioaf191.jpeg]
