# Supplementary material for: Identifying established human placental markers of schizophrenia in rodents after gestational ∆9-tetrahydrocannabinol exposure
Source: Biol Reprod. 2025 Aug 19;114(1):246–58. doi: 10.1093/biolre/ioaf191 (PMC12808552; doi:10.1093/biolre/ioaf191)
Supplement: Supplemental_Table_1_ioaf191 [file supplemental_table_1_ioaf191.docx]

| **Gene** | **Forward** | **Reverse** |
| --- | --- | --- |
| *Arpc3* | CCGACAAAATGCCGGCTTAC | CGGGTCCTTTGAACTGGCTT |
| *Atp2a2* | CGTGGAACCTTTGCCACTCAT | CAGGCTGCGCACACTCTTTA |
| *Atp5mk* | GTGATTCGGACGAAGAAGATTGA | CCAACAAAGCAATGCCTCCA |
| *Clk1* | ACAGCCGACGCTATGTTGAT | CCTTCGGTGACTCTTCCCAT |
| *Eif5* | AACATTCCAGAGGTCGCCAG | GACATAAACCCAACGCGGC |
| *Fes* | GCTCATCACTCACCTGCTGT | CTCTCCGAAGTTCCCCCTTC |
| *Furin* | CCTGATTGGGTTTCCCAGCA | GGGGCCAGATCCCCAGG |
| *Gid4* | CACGTGGACACTGGGAACTC | AGCCAGAAACTTGCCCCAG |
| *Iqgap1* | GAGGACGAGCTGCTGAAGATTA | AACATCTTGTTGGAAGCCGC |
| *Msi2* | CAGCACGACCCCGGTAAAAT | GCTATCTGGTGAGGTCTGCC |
| *Nt5c2* | CGGTGGGGCCGAATTCAT | GGTGATAGGCTTCTCGACGG |
| *Pappa2* | CAGCCATCATTGCAGGTGTG | GTCAGTGCGGAGGGAAAAGA |
| *Phf5a* | CAGGCTGGTGTAGCTATCGG | CATCAGAGACTCCGGGACCT |
| *Rccd1* | CAGTGAGAGAGGAAGCCACA | CCAGGGGAAGATCCAGGAGA |
| *Rsp10* | CGGCCTGCAAGATTCACAAG | CCGAAACCCCCTCTAAACTGG |
| *Snx3* | TGCGGCAGCTTCCTTTTAGA | GGATGACCAGCGACCTTGTT |
| *Trim8* | GCAGGACATTGAGGACCAGT | GCTCACTTTCTCCTCCACCA |
| *Vps33b* | TTCAGAACAGGGGCCATCCT | TCCTTTTTCCCAGGAAGCTGT |
| *Wbp1l* | GCTACATCTGTGACACGGGA | CCAGCCAGAACCACCAGAG |
| *B-actin* | CGCGAGTACAACCTTCTTGC | CGCAGCGATATCGTCATCCA |
| *Gapdh* | CTCTCTGCTCCTCCCTGTTC | CGATACGGCCAAATCCGTTC |

**Supplemental Table S1:** **Validated forward and reverse rat primers of all tested genes used for qPCR.**
