# Supplementary material for: Identifying established human placental markers of schizophrenia in rodents after gestational ∆9-tetrahydrocannabinol exposure
Source: Biol Reprod. 2025 Aug 19;114(1):246–58. doi: 10.1093/biolre/ioaf191 (PMC12808552; doi:10.1093/biolre/ioaf191)
Supplement: Supplemental_Table_2_ioaf191 [file supplemental_table_2_ioaf191.docx]

| **Gene** | **Forward** | **Reverse** |
| --- | --- | --- |
| *ATP5MK* | GCGGCTTTGGCAGATTGAAA | TGTGGCCAGTACACAGTTCA |
| *EIF5* | AGAACATTCCAGAGGTCGCC | GGATGAAGTGAGGCGCGATA |
| *FURIN* | GCCAACAACTATGGGACGCT | CTGACTGGACGTGAGGGTCT |
| *IQGAP1* | GTGAGGATGAGCTGCTGAAGATTA | TTGGTCTGTGGTAAGCTGGC |
| *RCCD1* | CGGGACTGAGCTCCTTGG | GGCTGGAGCTGCCTTATCTT |
| *RSP10* | GGCTGCTTCTCCCCAGGA | CTTCTTAGGCATCAACATCTGGC |
| *VPS33B* | TTGCGCTTCCCTGGGC | TTTCCAGGAAGCTGCTCCA |
| *B-ACTIN* | AAATCTGGCACCACACCTTC | AGAGGCGTACAGGGATAGCA |
| *PGK1* | AGAAGGCTGGTGGGTTTTTGA | GCAACTTTAGCTCCGCCCAG |

**Supplemental Table S2:** **Validated forward and reverse human primers of all tested genes used for qPCR.**
